# The interactions between cGAS-STING pathway and pathogens

**DOI:** 10.1038/s41392-020-0198-7

**Published:** 2020-06-10

**Authors:** Zhangliang Cheng, Tong Dai, Xuelin He, Zhengkui Zhang, Feng Xie, Shuai Wang, Long Zhang, Fangfang Zhou

**Affiliations:** 1 Institutes of Biology and Medical Science, Soochow University, 215123 Suzhou, P. R. China; 2 MOE Laboratory of Biosystems Homeostasis & Protection and Innovation Center for Cell Signaling Network, Life Sciences Institute, Zhejiang University, 310058 Hangzhou, China; 3 Kidney Disease Center, The First Affiliated Hospital, Zhejiang University School of Medicine, Hangzhou, China

**Keywords:** Innate immunity, Immunotherapy, Innate immunity, Immunotherapy

## Abstract

Cytosolic DNA is an indicator of pathogen invasion or DNA damage. The cytosolic DNA sensor cyclic guanosine monophosphate-adenosine monophosphate (cGAMP) synthase (cGAS) detects DNA and then mediates downstream immune responses through the molecule stimulator of interferon genes (STING, also known as MITA, MPYS, ERIS and TMEM173). Recent studies focusing on the roles of the cGAS-STING pathway in evolutionary distant species have partly sketched how the mammalian cGAS-STING pathways are shaped and have revealed its evolutionarily conserved mechanism in combating pathogens. Both this pathway and pathogens have developed sophisticated strategies to counteract each other for their survival. Here, we summarise current knowledge on the interactions between the cGAS-STING pathway and pathogens from both evolutionary and mechanistic perspectives. Deeper insight into these interactions might enable us to clarify the pathogenesis of certain infectious diseases and better harness the cGAS-STING pathway for antimicrobial methods.

## Introduction

Pathogen invasion triggers the host innate immune responses that initiate a series of activities to restrict pathogens and to sustain homeostasis. The detection of pathogens relies on germline-encoded pattern recognition receptors (PRRs), the ligands of which are called pathogen-associated molecular patterns (PAMPs). PAMPs are essential components of pathogens, and the activation of PRRs upon detecting PAMPs initiates the host’s defence to eliminate invading pathogens. As a key PAMP during infections, pathogen DNA that localises in abnormal cell sites, such as the cytosol and endosomes, alerts DNA sensors to trigger downstream innate immune responses.^1,2^ To date, multiple DNA sensors have been identified, among which cyclic guanosine monophosphate-adenosine monophosphate (cGAMP) synthase (cGAS) represents an essential one.^3,4^

cGAS is mainly localised in the cytosol and is activated once cytosolic DNA is detected. Activated cGAS then synthesises 2′,3′-cGAMP, which acts as an agonist for the endoplasmic reticulum (ER)-resident protein stimulator of interferon (IFN) genes (STING, also known as MITA, MPYS, ERIS and TMEM173).^5–11^ STING subsequently mediates several downstream signalling cascades, including those of autophagosome formation and production of a series of cytokines and chemokines, thus leading to potent antimicrobial responses.^10,12–15^ In recent years, the functional cGAS-STING axis that executes host defence activity has been identified in ancient species, indicating that the cGAS-STING pathway is an evolutionarily conserved defence mechanism against pathogens. Such an endless war has equipped both the host and invading pathogens with sophisticated and effective mechanisms to thwart each other, which also maximises their adaptability.

In this review, we present recent advances on the mechanisms underlying cGAS-STING signal transduction, as well as various inputs and outputs of this pathway. We further describe the origin and evolution of the cGAS-STING signalling and highlight its interactions with and counteractions to pathogens. Finally, we briefly summarise recent studies on the role of this pathway in cancers. We review the rapid growth of current knowledge on cGAS-STING signalling and discuss its potential contributions to antimicrobial drug design and antitumour therapy.

## Overview of the cGAS-STING signalling pathway

### cGAS: DNA sensor that synthesises cGAMP to stimulate STING

**Fig. 1:**
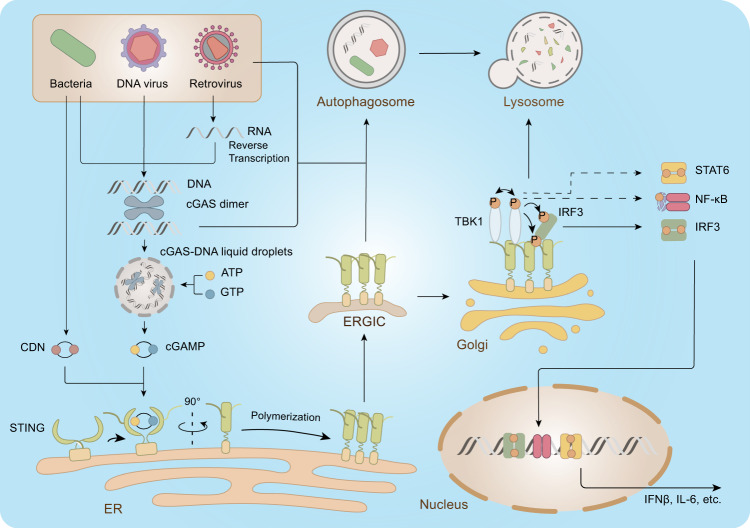
The cGAS-STING pathway. The presence of cytosolic DNA is an indicator of pathogen invasion. Cytosolic DNA is sensed by cGAS, resulting in the formation of cGAS-DNA liquid droplets, in which cGAS, ATP, and GTP are concentrated to powerfully enhance the production of cGAMP. STING binding to cGAMP undergoes conformational changes, leading to the release of C-terminal tails (CTT) and polymerization. Polymerized STING translocates from the ER to Golgi via ERGIC, where STING initiates the autophagy process, which contributes to the clearance of cytosolic DNA and pathogens. During the translocation process, STING also recruits TBK1. Recruited TBK1 undergoes trans-autophosphorylation and then phosphorylates STING in its CTT. Phosphorylated STING recruits IRF3 for phosphorylation and activation by TBK1. In addition to IRF3, TBK1 also activates NF-κB and STAT6. These activated transcriptional factors would translocate into the nucleus and induce the expression of various immunomodulatory genes, such as IFNβ and IL-6, leading to the establishment of an antipathogen state. After the translocation process, STING would be targeted to the lysosome for degradation to avoid overimmunization

The detection of cytosolic DNA by cGAS is the major input of the STING pathway in viral infection (Fig. 1). The cGAS binding to cytosolic DNA leads to its activation through conformational changes and dimerisation that result in a rearrangement of its catalytic site.^16,17^ This binding is independent of the DNA sequence but is dependent on DNA length.^18^ DNA of sufficient length is needed for cGAS dimers to associate with DNA in a cooperative manner, leading to the DNA-induced liquid-phase condensation of cGAS.^19^ The liquid droplets are likely to act as microreactors, in which the activated cGAS, ATP and GTP are enriched, thereby facilitating the synthesis of 2′,3′-cGAMP, which is a ligand for the STING dimer.

In its resting state, STING associates with the ER-resident protein stromal interaction molecule 1 (STIM1), which contributes to its retention in the ER.^20^ Binding to cGAMP disrupts the interactions between STING and STIM1, while enhancing those between STING and SEC24C, a component of the coat protein complex II (COPII), therefore initiating STING translocation from the ER to the Golgi apparatus via ER-Golgi intermediate compartment (ERGIC).^12,20–22^

Furthermore, binding to cGAMP triggers the release of the C-terminal tail (CTT) of STING and the polymerisation of STING dimers^23,24^ (Fig. 1). The released CTT recruits TANK-binding kinase (TBK1), whereas the polymerisation of STING dimers promotes trans-autophosphorylation and thus the activation of TBK1.^25–27^ Activated TBK1 phosphorylates the Ser residue in the pLxIS motif in STING CTT, which further recruits IFN regulatory factor 3 (IRF3). The recruited IRF3 is phosphorylated by TBK1 and then dimerises and translocates into the nucleus to promote type I IFN expression.^15^ The induced type I IFN contributes to the expression of a set of IFN-stimulated genes (ISGs) that executes antimicrobial functions, homing of immune cells and initiation of adaptive immune responses, leading to the establishment of antimicrobial immunity. Notably, while studies have shown that the translocation of STING is a prerequisite for inducing the STING-mediated type I IFN response,^21^ how intracellular trafficking and STING activation are coordinated is not well understood. Future studies are warranted to explore this interaction.

In addition to IRF3, the activated cGAS-STING pathway also promotes the transcription of nuclear factor kB (NF-κB) (Fig. 1). Although the detailed mechanisms remain unclear, the CTT of STING and its ER to Golgi translocation have been shown to be indispensable for inducing NF-κB signalling.^28,29^ While TBK1 activity has been reported to magnify NF-κB responses, it seems to be not essential for NF-κB activation.^28,30^ Moreover, signal transducer and activator of transcription 6 (STAT6) was reported to be recruited by STING for TBK1-mediated phosphorylation during viral infection. The activated STAT6 has thus contributed to the induction of a set of chemokines responsible for immune cell homing, thereby leading to reduced viral replication.^13^

In response to cGAMP, STING was recently found to translocate to the ERGIC, where it induces the lipidation of microtubule-associated protein 1 A/1B-light chain 3 (LC3), thus triggering autophagy to clear DNA and viruses in the cytosol through a mechanism independent of TBK1 activation and IFN induction.^12^ These findings revealed that autophagy induction via STING trafficking is likely to be a primordial function of the cGAS pathway.^12^ After traversing through the ERGIC/Golgi, STING would be targeted to lysosomes via autophagosomes for degradation.^12,31^ In summary, the cGAS-STING pathway utilises multiple downstream effectors to eliminate intracellular pathogens.

### Other inputs for STING activation

In addition to cGAS, other DNA sensors, including IFNγ-inducible protein 16 (IFI16), DEAD-box helicase 41 (DDX41), DNA-dependent protein kinase (DNA-PK), and heterogeneous nuclear ribonucleoprotein A2B1 (hnRNPA2B1), also mediate downstream signalling through STING.^32–35^ The existence of various DNA sensors seems to be a supplement to cGAS for pathogen detection in certain contexts.^35,36^ Alternatively, these redundant signalling mechanisms may represent a strategy for the host to counter the pathogen’s evasion from certain DNA sensors.

Apart from the endogenous 2′,3′-cGAMPs produced by cGAS, other cyclic dinucleotides (CDNs), including c-di-AMP, c-di-GMP and 3′,3′-cGAMP, which are produced directly from bacteria, can induce the activation of STING through bypassing DNA sensors, as well.^23,37–39^ However, due to the different cell contexts and/or distinct, although similar, STING conformational changes induced by different agonists, the consequence of STING activation is likely to be diverse.^23,40^

In addition, STING has also been reported to interact with the RNA sensor retinoic acid-inducible gene 1 (RIG-I) and the downstream adapter mitochondrial antiviral signalling protein (MAVS, also known as VISA, IPS-1 and Cardif), which are responsible for viral RNA sensing.^10,14,41^ Because the deletion of STING impairs RIG-I-mediated innate signalling, STING may play a role in defending against RNA viruses. Furthermore, virus-cell fusion has been reported to activate STING in a DNA-independent manner; however, its detailed mechanisms are currently unknown.^42^ Overall, these results imply several inputs converging on the cGAS-STING pathway, suggesting the essential role of STING activation in monitoring cellular contexts.

## Evolution of the cGAS-STING pathway

The majority of the work regarding the cGAS-STING signalling pathway is focused on mammalian cell lines. However, the primary sequence homologs of both cGAS and STING have been identified in *Monosiga brevicollis*, which is considered as the closest living relative of animals.^43^ More strikingly, one recent study has identified a role for the bacterial cGAS-like enzyme dinucleotide cyclase in *Vibrio* (DncV) in mediating antiviral defence, and *DncV* is located in the same operon as its effector gene, which encodes cGAMP-activated phospholipase in *Vibrio* (CapV). Upon phage infection, DncV is triggered to produce 3′,3′-cGAMP, which acts as an agonist for phospholipase. The activation of phospholipase results in bacterial membrane degradation and cell death, thereby preventing further infection and propagation of the phage.^44,45^

Notably, in some bacteria and primitive eukaryotes, the effector gene in the potential anti-phage operon contains a Toll-interleukin (IL) receptor (TIR) domain, instead of a phospholipase domain, and a STING domain, although how this operon executes its function is unclear.^45^ Overall, these results suggest that the origin and antimicrobial functions of cGAS and STING span far beyond the mammals and may even predate the phylogeny of animals. The prolonged combats between the cGAS-STING pathway and pathogens have driven the rapid evolution of both cGAS and STING.

Recent studies that focused on the cGAS-STING pathways in nonmammalian species and their comparison between different species have already shed evolutionary insights on this topic. Perspectives from the evolutionary viewpoint would provide us with a deeper understanding of how the modern cGAS-STING signalling response is shaped, as well as comprehensive insights on the continuous arms race between hosts and pathogens.

### cGAS and STING in invertebrates

**Fig. 2:**
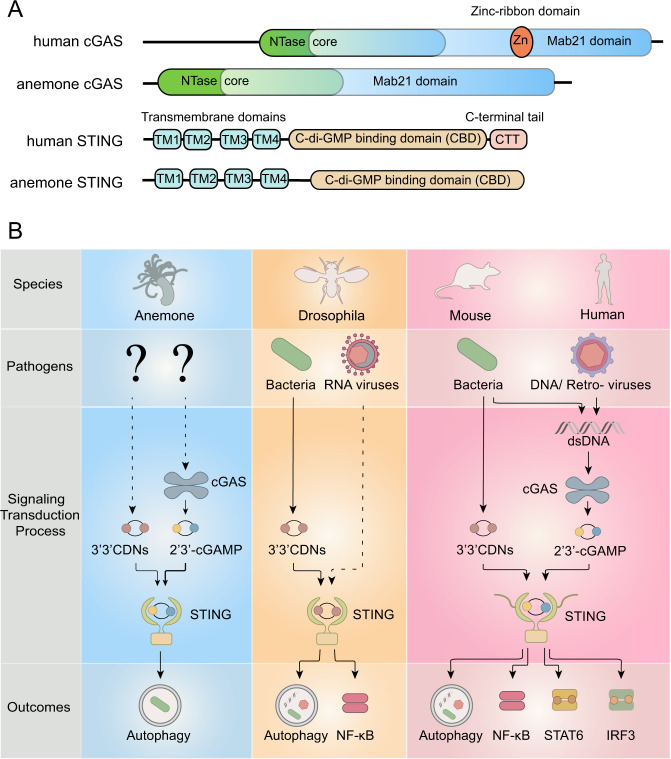
Evolution of the cGAS-STING pathway. **a** Comparison of the functional domains in cGAS and STING between invertebrate (anemone) and vertebrate (human) species. Compared with human cGAS, anemone cGAS has a shorter N terminal and lacks the zinc-ribbon finger, both of which are involved in DNA binding in vertebrate cGAS. The C-terminal tail, which is essential for IFN induction in vertebrate STING, is also absent in anemone STING. **b** Currently identified cGAS-STING pathway in different species. While the cGAS-STING pathways in different species share a similar framework, there are two notable observations: firstly, no studies have suggested that invertebrate cGAS could detect DNA as vertebrate cGAS do, and the function of invertebrate cGAS remains unclear; secondly, the cGAS-STING pathway seems to have acquired more antipathogen methods during evolution

Bioinformatic analyses of cGAS and STING homologs have revealed their wide distribution across animal species, as well as their significant sequential differences.^43^ Compared to vertebrate cGAS, that of invertebrates lacks the zinc-ribbon domain in its C-terminal and has a reduced N-terminal length, positing its inability to bind DNA. Furthermore, the CTT of STING, which is essential for downstream type I IFN signalling induction in vertebrates, is absent in invertebrates^43^ (Fig. 2a). Considering that IFN genes have only been identified in vertebrates, it is reasonable to infer that the invertebrate STING is unable to induce type I IFN signalling.^46^

The characteristics of invertebrate cGAS and STING suggested by bioinformatic analyses have been corroborated by biochemical and genetic assays. The existence of the functional cGAS-STING axis has been confirmed in *Nematostella vectensis*, an ancient anemone species that has diverged from humans more than 500 million years ago^12,47^ (Fig. 2b). However, the cGAS-STING axis in *N. vectensis* is much different from that in mammals. Firstly, *N. vectensis* cGAS (nv-cGAS) is not activated by double-stranded DNA (dsDNA), and its agonist remains elusive. Secondly, *N. vectensis* STING (nvSTING) exhibits a remarkably enhanced affinity for 3′,3′-cGAMP and 3′,3′-c-di-GMP compared to human STING (hSTING). Lastly, nvSTING expressed in human cells could not activate the IFN signalling pathway but could induce autophagy, which might suggest the original function of STING.^12,47^

While the physiological function of cGAS and STING in *N. vectensis* remains elusive, recent studies on *Drosophila* have revealed an indispensable role of STING in antimicrobial immunity (Fig. 2b). Following infection by *Listeria monocytogenes*, *Drosophila melanogaster* STING (dmSTING) detected CDNs produced by bacteria and mediated the induction of antimicrobial peptides through the NF-κB factor Relish, thus reducing *Listeria*-induced lethality.^48^ In this study, by exogenously expressing it in mammalian cells, researchers found that dmSTING was unable to activate IRF3, suggesting that, in addition to autophagy, NF-κB induction might be another original function of STING predating its ability to induce IFN signalling. In other independent studies, dmSTING was proved to protect the host from infections by RNA viruses via autophagy and/or activation of NF-κB.^49,50^ However, how dmSTING is activated during viral infection is currently unclear. Mutating the dmSTING residues R232 and F234, which correspond to residues involved in CDN binding in hSTING, abrogated the antiviral activity of dmSTING, indicating that CDNs may act as agonists for STING upon viral infection.^50^

Despite the obvious participation of dmSTING in Drosophila immunity, to date, no study has identified a role for cGAS in the immunity of this organism. The presence or absence of cGAS orthologs seems to make no difference in the mortality rates resulting from infections by *Listeria* or DNA viruses, such as invertebrate iridescent virus 6 (IIV6), in *Drosophila*.^48^ Therefore, the exact function of cGAS orthologs in *Drosophila*, as well as how cGAS has gained its role in antiviral immunity throughout evolution, remains to be determined.

### cGAS and STING in vertebrates

While the structural domains of cGAS and STING are conserved in vertebrates, both proteins are subjected to recurrent positive selections.^51,52^ The sites under positive selection in cGAS are mainly located at the protein surfaces or regions that contact with DNA, at least in the case of primates.^51^ In hSTING, positive selection occurs in areas that affect its CDN binding affinity.^52^ The hotspots of positive selection in these two proteins are consistent with their marked roles in host-pathogen interaction.^53^ Among all cGAS and STING homologs in vertebrates, the distinct features between those in humans and mice are the most intensively studied.

The divergence in ligand selectivity between mouse cGAS (m-cGAS) and human cGAS (h-cGAS) is notable. Compared to m-cGAS, h-cGAS exhibits greater preference to long dsDNA.^18,54^ The increased number of basic residues in the so-called site-C, which is a newly identified DNA-binding interface in the catalytic domain of cGAS, of h-cGAS contributes to the multivalence of this protein, facilitating liquid-phase condensation and enhancing enzymatic activity upon the detection of long dsDNA.^54^ In addition, substitutions of N172/R180 in m-cGAS to K187/L195 in h-cGAS weaken a portion of the cGAS-DNA-binding surface that is necessary during the recognition of short dsDNA but is dispensable upon detection of long dsDNA. Thus, this further enhances the preference of h-cGAS to long dsDNA.^55^ Another consequence of human-specific N187K/R195L substitutions is the impaired enzyme activity of h-cGAS, which may be a compensation for the increased sensitivity to long dsDNA to avoid excessive immune responses.

As is the case of cGAS, hSTING and mouse STING (mSTING) also exhibit dramatically distinct ligand selectivities. Compared to mSTING, hSTING shows greater preference for 2′,3′-cGAMP than for 3′,3′-cGAMP or c-di-GMP.^23,56^ Furthermore, while DMXAA (also known as Vadimezan or ASA404), a drug developed for antiviral or antitumour therapies, works well as a ligand for mSTING, it fails to target hSTING.^40,57^ The high selectivity of hSTING may largely stem from the high activation energy for transition between open and close conformation, which may be responsible for preventing ligands from slipping out of the binding sites.^57^ Thus, an effective activator of hSTING must bind it with enough favourable interactions to stabilise its close conformation and achieve high affinity.

The overall consequence of evolutionary divergences between human and mouse cGAS and STING homologs renders the human cGAS-STING pathway more sensitive to long dsDNA, as well as blind to CDNs produced by bacteria and short cytosolic dsDNA, which might result in the development of autoimmune diseases. While the adaptive significance of these divergences is hard to interpret, it may be related to the distinct spectra of pathogens that infect mice and humans, as well as the different immune strategies that they adopt.

Although the CTTs of hSTING and mSTING conserve their role in recruiting TBK1 and IRF3 and in triggering downstream IFN signalling, their function and structure have diversified to a great extent during vertebrate evolution. As the main window of the output of the STING signalling pathway, the plasticity exhibited by the CTT of STING contributes to the malleability of the whole signalling pathway. In bats, the replacement of a conserved S358 in CTT, which is required for IRF3 binding, dampens STING-dependent IFN activation and renders bats tolerant to viruses or flight-induced cytosolic DNA.^58^ In zebrafish, activated STING induces robust NF-κB downstream signalling, in contrast to the main IFN signalling induced by mSTING or hSTING.^29^ Researchers have identified a module appending to the end of the CTT of zebrafish STING that is responsible for triggering downstream NF-κB signalling. Intriguingly, appending this motif to the CTT of mSTING endows mSTING with the ability to induce an additional set of NF-κB-dependent genes besides the canonical IFN-dependent genes, suggesting a modular feature of CTT.^29^ Notably, the whole CTT could also be perceived as a module. Simply appending the CTT to oligomerising platforms enables researchers to design various nanomachines that are able to induce IFN responses when receiving the corresponding input signals.^59^ The modular feature of the CTT lowers the evolutionary barrier to transform STING downstream signalling and raises the speculation that the sudden appearance of the CTT in vertebrate STING is the result of obtaining the module.

Overall, mutations under selective pressure and modular features help shape and reform the input and output of the cGAS-STING pathway during evolution, thus contributing greatly to the plasticity of this pathway. Its plasticity is indispensable for the host’s ability to antagonise the continuously evolving pathogen-encoded evasion mechanisms and directly contributes to the development of strategies between the cGAS-STING pathway and pathogens. In the following sections, we will discuss these interactions arising from the long course of evolution.

## Multiple pathogen detection strategies

**Fig. 3:**
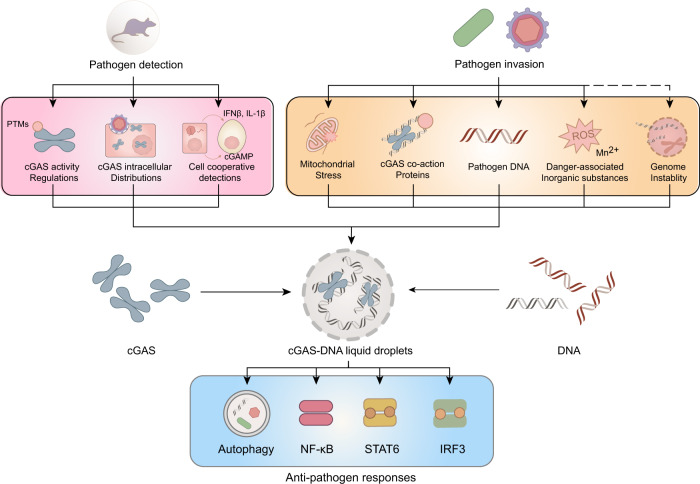
Multiple detection strategies against pathogens. The dynamic regulations of cGAS activity, the wide intracellular distributions of cGAS, and the cell cooperative detection of pathogens constitute several layers of pathogen detection. In addition to the presence of PAMPs, other information indicating pathogen invasion, including the activation of cGAS coaction proteins and the emergence of danger-associated signals, can be integrated into pathogen detection. Multilayered pathogen detection and the capacity of integrating various information render the cGAS-STING pathway with unique sensitivity to infection to initiate a series of antipathogen responses

The prerequisite for initiating innate immunity against pathogens is the sensitive detection of pathogen infections. Therefore, to evade the surveillance of the cGAS-STING pathway, a common microbial response consists of hiding their DNA from cGAS.^60^ To antagonise the evasion of pathogens, multiple detection strategies have been adopted (Fig. 3).

### Localisation of cGAS

The basic detection strategy consists of distributing cGAS in places where it can encounter pathogen DNA while avoiding self-DNA detection. Besides the cytosol, recent studies have reported that cGAS is also localised at the plasma membrane and in the nucleus.^61,62^ Plasma membrane-resident cGAS might represent a strategy to avoid self-DNA detection, given that the presence of cytosolic nucleases would limit the diffusion of cellular self-DNA to the plasma membrane region under resting state. Viral infection would provide cGAS with sufficient agonists and release cGAS from the plasma membrane to facilitate its signalling in the cytosol.^61^ The nucleus-localised cGAS might facilitate the detection of viruses that only expose their DNA in the nucleus, and the nucleosome structure of self-DNA and special localisation of cGAS in the nucleus might enable cGAS to discriminate self-DNA from viral DNA.^62–64^ Thus, the intracellular distribution of cGAS provides surveillance that covers sites where pathogens release their DNA while trying to avoid aberrant self-DNA detection.

### Self-DNA detection

After the encounter with DNA, only when the DNA concentration reaches a certain threshold can the liquid-phase condensation of cGAS be initiated, leading to the robust activation of the cGAS-STING pathway.^19^ Both host DNA and pathogen DNA have the potential to be detected by cGAS, thereby jointly contributing to reaching the concentration threshold. Therefore, to avoid the continuous activation of the cGAS-STING pathway in resting cells, multiple strategies are adopted to limit self-DNA detection, such as restriction of DNA or cGAS activity and compartmentalisation.^65^ However, upon pathogen infection, the steady state is disrupted, and self-DNA detection contributes to the activation of the cGAS-STING pathway, enabling the indirect detection of pathogen infection. For example, the herpes simplex virus type 1 (HSV-1) infection has been shown to result in mitochondrial DNA (mtDNA) dysregulation, leading to the cytosolic presence of mtDNA, which is necessary for fully engaging the antiviral innate immunity in a cGAS-STING-dependent manner.^66^ In addition, cells can initiate the cGAS-STING pathway against the dengue virus, an RNA virus, through self-mtDNA detection, as well.^67^ Intriguingly, a protein encoded by the dengue virus has been shown to target cGAS for degradation, which suggests the evolutionary pressure on pathogens to antagonise self-DNA sensing.^68^ Furthermore, increased self-DNA detection and enhanced antimicrobial innate immunity can also result from genome instability.^69,70^ Nevertheless, whether genome instability is an additional pathogen sensory mechanism in the context of infection remains to be determined.

### The key node: interaction between DNA and cGAS

Various proteins indicating infection enhance the interaction between DNA and cGAS, thus lowering the DNA detection threshold and increasing the sensitivity of cGAS. For example, the presence of the mitochondrial transcription factor A (TFAM) and mitochondrial nucleoid proteins HU in the cytosol, which indicate mitochondrial stress and bacterial infection, respectively, prearranges DNA in a structure suitable for cGAS binding.^18^ In cells infected by human immunodeficiency virus (HIV) type 2, non-POU domain-containing octamer-binding protein (NonO) is able to detect and bind viral capsid proteins, placing HIV-2 DNA in the proximity of cGAS and enhancing their interaction.^64^ In addition, changes in the cell’s intrinsic environment may facilitate DNA detection. It has been reported that the elevated cytosolic Mn^2+^ concentration resulting from cytoplasmic acidification induced by viral infection and from the disrupted mitochondrial membrane potential contributes to the increased sensitivity of cGAS to dsDNA.^71^ Reactive oxygen species (ROS) produced by macrophages and neutrophils to antagonise pathogens have been suggested to induce oxidative modifications in the DNA that would be resistant to nuclease degradation, thus leading to its enhanced detection by cGAS.^72^ Other strategies that play a role in facilitating the detection of pathogen infections via the cGAS-STING pathway, including utilising signals delivered by other cells and post-translational modifications (PTMs) of cGAS and STING, will be discussed next. Overall, the wide distribution of DNA detectors in cells and their ability to integrate multiple signals largely contribute to the sensitivity of the cGAS-STING pathway.

### Establishment and amplification of immunity in bystander cells

Alarming signals transduced by infected cells can prepare or activate the cGAS-STING pathway in bystander cells for countering pathogens. Cytokines, such as IFNβ, IFNα and IL-1β, are an important category of such alarming signals. In cells stimulated by type I IFN, the transcriptional levels of both cGAS and STING build up, leading to elevated protein levels.^73,74^ Thus, a more sensitive and robust reaction can be initiated when cells encounter pathogens. IL-1β promotes cell-intrinsic immune protection in bystander cells as well, but in a different way. IL-1β signalling causes mitochondrial stress and mtDNA release, leading to the activation of stress-induced (but not pathogen-induced) cGAS-STING signalling.^75^ In addition, if cGAS is activated in infected cells, synthesised cGAMP might be transferred horizontally and might serve as an alarming signal. Compared to other alarming signals, cGAMP acts exclusively in the cGAS-STING pathway, which may prevent signal distortion. In densely packed tissues, membrane fusion or gap junctions may represent major strategies to transfer cGAMP, while enveloping cGAMP in the virus may be a way to alert more distant cells.^76–79^ In addition, a recent study has identified a clear role for volume-regulated anion channels (VRAC) in transporting 2′,3′-cGAMP into bystander cells during HSV-1 infection, and the activation of VRAC is enhanced by inflammatory factors, such as IL-1β and TNF, associated with the viral infection, therefore suggesting another strategy for intercellular cGAMP transfer during infection.^80^ Besides, the folate transporter solute carrier family 19 member 1 (SLC19A1) present in human cell lines may also serve as a potential mediator of intercellular transportation of cGAMP in immune responses.^81,82^ The ability of the cGAS-STING pathway to establish and amplify immunity in bystander cells provides the host with key advantages.^76^ Immunity in bystander cells not only represents a cell cooperation-based pathogen detection mechanism, but also allows the full establishment of an antiviral stage in cells that are free of virus-encoded inhibitory mechanisms.

## Post-translational control of the cGAS-STING pathway

**Fig. 4:**
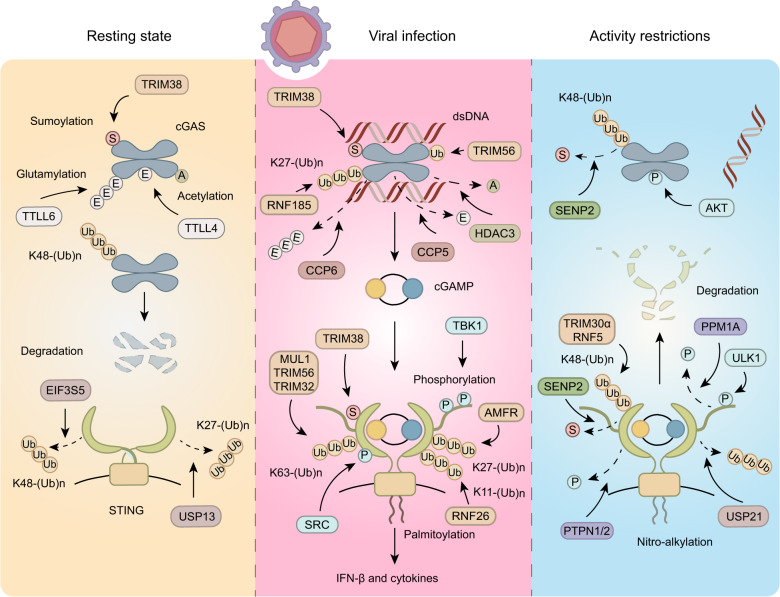
Post-translational modifications of cGAS and STING. This figure illustrates the post-translational modifications of cGAS and STING in resting states upon viral infection, which serve to restrict the activity of the cGAS or STING after activation. A acetylation, E glutamylation, P phosphorylation, Ub ubiquitination, S sumoylation, K11-(Ub)n K11-linked polyubiquitination, K27-(Ub)n K27-linked polyubiquitination, K48-(Ub)n K48-linked polyubiquitination, K63-(Ub)n K63-linked polyubiquitination

The strategies presented above ensure the effective detection of pathogens, while the liquid-phase condensation of cGAS, participation of the second messenger, and polymerisation of STING serve as powerful ways to amplify immune signals to eliminate pathogens. However, given the potential detrimental consequence of excessive immunity, the tight and dynamic control of the cGAS-STING signalling pathway is needed to sustain the delicate balance between the elimination of pathogens and the prevention of harming the host. PTMs, which refer to covalent modifications of proteins, participate in the control of each step of the cGAS-STING signalling cascades and are crucial to their dynamic regulation (Fig. 4).

In resting cells, PTMs help sustain steady states. Several modifications of cGAS, such as glutamylation, acetylation, sumoylation and ubiquitination, have been observed in unstimulated cells (Table 1). The monoglutamylation exerted by tubulin tyrosine ligase-like 4 (TTLL4) impairs the synthase activity of cGAS, and the polyglutamylation catalysed by TTLL6 impedes the DNA-binding affinity of cGAS.^83^ cGAS also undergoes acetylation at K384, K394 or K414, which are vital modifications to keep cGAS inactive, without DNA challenge or viral infection, and aspirin prevents self-DNA-induced autoimmunity by efficiently acetylating cGAS.^84^ In addition to suppressing the activity of cGAS, certain PTMs maintain the protein levels at a proper range. Sumoylation at K217 and acetylation at K414 antagonise K48-linked ubiquitination, thereby stabilising cGAS and ensuring that immune responses are initiated on time.^84,85^ The deubiquitinating enzyme (DUB) ubiquitin-specific protease 13 (USP13) has been reported to deconjugate K27-linked polyubiquitin chains from STING, resulting in a suppressed basal STING activity.^86^ However, the E3 ligase that co-regulates the basal STING activity has not yet been identified.

**Table 1:** Post-translational modifications of cGAS and STING

Protein	Modification	Residues	Enzyme	Occurring contexts	Functions	Refs
cGAS	Monoubiquitination	K335	TRIM56	Stimulated cells	Promotes cGAS dimerization and DNA-binding activity.	^83^
	K27-linked polyubiquitination	K173/K384	RNF185	Stimulated cells	Promotes enzymatic activity of cGAS	^84^
	K48-linked polyubiquitination	At least at K285/K479	N.D.	Resting cells and stimulated cells	Facilitates degradation of cGAS in a proteasome pathway	^81^
	Removal of K48-linked polyubiquitination	K414	USP14	Stimulated cells	Stabilizes cGAS	^98^
	Phosphorylation	S305	AKT	Stimulated cells	Impairs enzymatic activity of cGAS	^94^
	Polyglutamylation	E272	TTLL6	Resting cells	Impairs DNA-binding ability of cGAS	^79^
	Removal of polyglutamylation	E272	CCP6	Stimulated cells	Reverse inhibitory modification	^79^
	Monoglutamylation	E302	TTLL4	Resting cells	Impairs enzymatic activity of cGAS	^79^
	Removal of monoglutamylation	E302	CCP5	Stimulated cells	Reverse inhibitory modification	^79^
	Acetylation	K384/K394/K414	N.D.	Resting cells	Keeps cGAS in inactive states	^80^
	Removal of acetylation	K384	HDAC3	Stimulated cells	Reverse inhibitory modification	^80^
	Sumoylation	K231	TRIM38	Resting cells	Stablizes cGAS and impairs DNA-binding ability of cGAS	^81^
	Sumoylation	K479	TRIM38	Stimulated cells	Stabilizes cGAS	^81^
	Removal of sumoylation	K479	SENP2	Stimulated cells	Facilitates degradation of cGAS	^81^
STING	K11-linked polyubiquitination	K150	RNF26	Stimulated cells	Stabilizes STING	^85^
	K27-linked polyubiquitination	K137/K150/K224/K236	AMFR	Stimulated cells	Promotes recruitment of TBK1	^86^
	Removal of K27-linked polyubiquitination	N.D.	USP13	Resting cells and stimulated cells	Prevents recruitment of TBK1	^82^
	Removal of K27-linked polyubiquitination	N.D.	USP21	Stimulated cells	Inhibits the formation of STING –TBK1-IRF3 complex	^97^
	K63-linked polyubiquitination	K20/K150/K224/K236	TRIM32	Stimulated cells	Promotes interaction with TBK1	^88^
	K63-linked polyubiquitination	K224/K236/K289/K338	MUL1	Stimulated cells	Promotes dimerization and trafficking of STING	^89^
	K63-linked polyubiquitination	K150	TRIM56	Stimulated cells	Promotes dimerization of STING and recruitment of TBK1	^87^
	Removal of K63-linked polyubiquitination	N.D.	USP21	Stimulated cells	Inhibits the formation of STING-TBK1-RF3 complex	^97^
	K48-linked polyubiquitination	K275	TRIM30α	Stimulated cells	Promotes degradation of STING in a proteasome pathway	^100^
	K48-linked polyubiquitination	K150	RNF5	Stimulated cells	Promotes degradation of STING in a proteasome pathway	^99^
	Removal of K48-linked polyubiquitination	N.D.	USP20	Stimulated cells	Stabilizes STING	^102^
	Removal of K48-linked polyubiquitination	N.D.	CYLD	Stimulated cells	Stabilizes STING	^101^
	Removal of K48-linked polyubiquitination	N.D.	EIF3S5	Stimulated cells	Stabilizes STING	^103^
	Phosphorylation	Y245	SRC	Stimulated cells	Enhances the activation of STING	^92^
	Phosphorylation	S358	TBK1	Stimulated cells	Facilitates aggregation of STING	^91^
	Phosphorylation	S366	TBK1	Stimulated cells	Facilitates recruitment of IRF3	^13^
	Phosphorylation	S366	ULK1	Stimulated cells	Facilitates degradation of STING	^104^
	Dephosphorylation	Y245	PTPN1/2	Stimulated cells	Promotes degradation of STING in a proteasome pathway	^96^
	Dephosphorylation	S358	PPM1A	Stimulated cells	Impairs STING aggregation	^91^
	Palmitoylation	C88/91	DHHC	Stimulated cells	Enhances type I Interferon responses	^93^
	Nitro-alkylation	C88/C91/H16	N.D.	Stimulated cells	Antagonizes palmitoylation and impairs STING signaling	^95^
	Sumoylation	K338	TRIM38	Stimulated cells	Promotes oligomerization and prevents degradation of STING	^81^
	Removal of sumoylation	K338	SENP2	Stimulated cells	Facilitates degradation of STING	^81^

*N.D.* not determined

In response to stimuli, the interactions of cGAS with histone deacetylase 3 (HDAC3), cytosolic carboxypeptidase 5 (CCP5), and CCP6 are enhanced, leading to the removal of inhibitory acetylation, monoglutamylation and polyglutamylation, respectively.^83,84^ Meanwhile, the monoubiquitination exerted by tripartite motif-containing 56 (TRIM56) and K27-linked polyubiquitination at K384 exerted by ring finger protein 185 (RNF185), occuring in residues that were previously occupied by inhibitory acetylation, result in enhanced sensing and enzymatic activity of cGAS.^84,87,88^ Furthermore, sumoylation catalysed by TRIM38 at K464 of m-cGAS (K479 being the corresponding residue in h-cGAS) stabilises it during early infection by preventing K48-linked polyubiquitination at the same lysine residue. Suitable PTMs are also essential for STING to properly execute its function upon pathogen evasion. The released CTT of STING, occurring after binding to cGAMP, has been shown to be sumoylated by TRIM38, leading to enhanced activation and stabilisation of STING.^85^ Mechanistically, the sumoylation of STING facilitates its oligomerisation, which is essential for downstream IRF3 activation, and masks an adjacent motif recognised by heat-shock cognate protein 70 kDa (HSC70), which in turn mediates the degradation of STING via chaperone-mediated autophagy (CMA).^85^ In addition, STING undergoes several forms of ubiquitination to sustain or boost its activation (Table 1). K11-linked polyubiquitination targeted by RNF26 competes with K48-linked ubiquitin chains at K150 to balance proper protein levels of STING after viral infection.^89^ The K27- and K63-linked polyubiquitination of STING was shown to potentiate TBK1 recruitment and downstream signalling activation. K63-linked polyubiquitination at K224, which is catalysed by mitochondrial E3 ubiquitin protein ligase 1 (MUL1), was suggested to be the predominant ubiquitination type in STING required for its trafficking and is prerequisite for its subsequent phosphorylation and degradation.^90–93^ During trafficking, STING phosphorylation at S366 (S365 being the corresponding residue in mSTING) by TBK1 was shown to be critical for inducing IRF3-mediated downstream IFN signals as mentioned above.^15,26,27,94^ The phosphorylated STING interacts with a positively charged phosphor-binding domain of IRF3, thereby recruiting IRF3 for TBK1 phosphorylation and activation.^15^ Consistently, the S366A mutation in STING has been reported to be unable to interact with and activate IRF3 upon DNA stimulation. Studies have identified two additional phosphorylated sites in STING at Y245 and S358 that fuel its activation.^95,96^ In addition, STING palmitoylation occurring in the TGN (trans-Golgi network) is required for type I IFN responses, which was hypothesised to be important to STING clustering.^97^ However, a recent study has disproved this hypothesis by showing that the polymerisation of STING occurs at the ER.^23^ Therefore, the real function of palmitoylation needs to be further investigated.

While various PTMs participate in the activation of the cGAS-STING pathway, some restrict this activation to prevent untoward consequences. For example, cGAS is phosphorylated by AKT at S305 after being activated for a while, leading to a suppressed enzymatic activity.^98^ Nitro-fatty acids synthesised endogenously in response to viral infection covalently modify STING and inhibit its palmitoylation, and thus activation.^99^ In addition to adding negative PTMs, the removal of positive PTMs represents a way to restrict the cGAS-STING signalling. cGAS and STING are desumoylated by SUMO-specific protease 2 (SENP2) at the late phase of infection, leading to their degradation.^85^ Protein phosphatase 1 A (PPM1A) and protein tyrosine phosphatase non-receptor type 1/2 (PTPN1/2) reportedly dephosphorylate S358 and Y245 in STING, respectively, therefore attenuating its activation.^95,100^ Moreover, USP21 is activated upon prolonged DNA virus stimulation and then hydrolyses K27- and K63-linked polyubiquitin chains on STING at a later stage.^101^ PTMs that trigger the degradation of cGAS or STING may be one of the most exhaustive ways to restrict immune signalling. It has been shown that K48-linked polyubiquitination of cGAS facilitates cGAS degradation via the p62-mediated autophagy or proteasome pathway.^85,102^ The E3 ubiquitin ligase RNF5 and TRIM30α interact with STING and catalyse its polyubiquitination with K48-linked polyubiquitin chains after viral infection. This modification triggers STING degradation through the proteasome pathway and diminishes downstream antiviral signalling.^103,104^ Conversely, the K48-linked polyubiquitination of STING is reversed by various DUBs, including CYLD, USP20 and eukaryotic translation initiation factor 3 subunit 5 (EIF3S5)^105–107^ (Table 1). Therefore, the subtle balance between the levels of E3 ligase and DUBs regulates the active cGAS-STING pathway.

Intriguingly, the phosphorylation of STING at S366 by unc-51-like autophagy activating kinase 1 (ULK1) and K63-linked polyubiquitination of STING, which have been mentioned above to activate STING, have been reported to also mediate the degradation of STING.^31,108^ Although these inconsistencies have not been clearly explained yet, it is possible that the function of certain PTMs depends on modification events. For example, the phosphorylation of mSTING at S365 leads to the recruitment of SENP2, which facilitates the degradation of mSTING, as mentioned above.^85^ Moreover, while TBK1 is able to mediate the subsequent phosphorylation and activation of IRF3 after phosphorylating S366 in STING, ULK1 is not.^85^ Therefore, the consequence of S366 phosphorylation catalysed by TBK1 and ULK1 appears to exert distinct functions.

To date, although multiple enzymes and their corresponding PTMs have been identified to covalently modify cGAS or STING (Table 1), little is known about how different PTMs crosstalk to each other and how the activities of enzymes that execute PTM are properly regulated during infection. In addition, the detailed mechanisms underlying the regulations of cGAS and STING by most PTMs are not clearly understood. Moreover, whether or how PTMs regulate the sensitivity of the cGAS-STING pathway towards different inputs and the directions of this pathway towards different outputs is largely unknown. Further studies are needed to provide deeper insights on this dynamic regulatory process.

## Pathogen evasion from the cGAS-STING pathway

Given the strong selective pressure imposed by the cGAS-STING pathway to pathogens, especially viruses, it is not surprising to find some pathogens that can successfully establish infection states in the host and that possess effective strategies to counter or escape the surveillance of the cGAS-STING pathway. In this paper, we will introduce the two main categories of pathogen evasion: inhibition of the signal transduction process and avoidance of DNA exposure.

### Inhibition of the signal transduction process

**Fig. 5:**
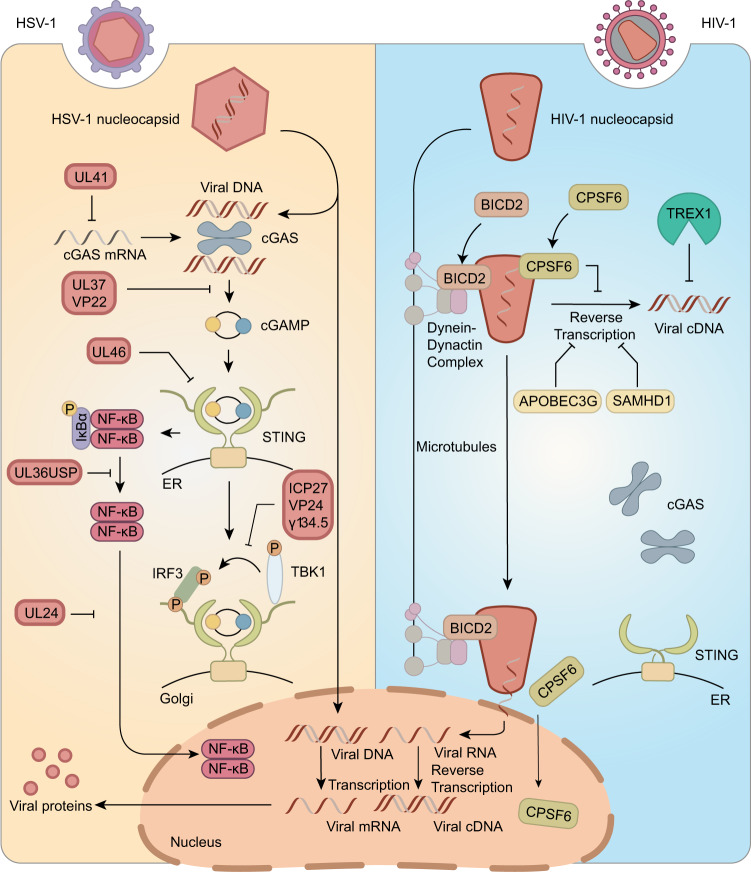
Pathogen evasions from cGAS-STING pathway. Different strategies are adopted by HSV-1 and HIV-1 to evade from the surveillance of the cGAS-STING pathway. Whereas HSV-1 mainly encodes a variety of proteins to counter key signal transduction processes of the cGAS-STING pathway, HIV-1 utilizes cellular autonomous restriction factors and transport systems to limit exposure of its viral DNA to cGAS. The viral proteins encoded by HSV-1 are shown in the same colour as HSV-1 capsids

By inhibiting the signal transduction of the cGAS-STING pathway, microbes are protected from host antimicrobial defence. This strategy is well illustrated in HSV-1, a DNA virus possessing high capacity of evading host immunity (Fig. 5).

Because its invasion can be detected by cGAS, numerous viral proteins encoded by HSV-1 negatively modulate cGAS as a countermeasure, thus attenuating the activation of the cGAS-STING pathway. For instance, UL41 selectively degrades cGAS mRNA via its RNase activity, leading to a reduced protein level of cGAS and an abrogated detection of viral DNA.^109^ cGAS is also regulated by viral proteins. The HSV-1 VP22 interacts with cGAS and dampens its enzymatic activity through an unknown mechanism.^110^ Furthermore, the HSV-1 tegument protein UL37 was demonstrated to deamidate an essential Asn residue in human and mouse cGAS, leading to an impaired cGAS activity.^111^ Interestingly, this critical Asn is not conserved in the cGAS of many non-human primates, thus providing an example of species-specific host-pathogen interactions.^111^

The regulation of STING by viral proteins seems to be contradictory. While HSV-1 UL46 has been suggested to negatively regulate STING protein levels, infected cell protein 0 (ICP0), ICP4 and US3 protein kinase (US3-PK) encoded by HSV-1 have been reported to stabilise STING.^112,113^ In addition, the role of STING in HSV-1 infection is elusive and dependent on the cell type. In cancer-derived HeLa cells or HEp-2 human laryngeal carcinoma cells, STING was proposed to facilitate HSV-1 production via an unknown mechanism, whereas in human embryonic lung cells or HEK293T cells derived from normal tissues, STING reduces viral yields.^112^ Whether the virus adopts different regulation strategies towards STING in different cell types to achieve maximum colonisation is an interesting topic that remains to be explored.

Finally, as the major effectors of the cGAS-STING pathway and regulators of innate immunity responses, IRF3 and NF-κB are intensively regulated by viral proteins. For example, HSV-1 ICP27 has been reported to interact with the STING signalosome in a manner dependent on TBK1 activity, leading to reduced phosphorylation and impaired activity of IRF3.^114^ In addition, VP24 and γ_1_34.5 disrupt the interaction of TBK1 and IRF3, thus impairing IRF3-mediated transcription.^115,116^ As for NF-κB, HSV-1 UL36USP was shown to stabilise IκBα, an inhibitor of NF-κB. UL24 encoded by HSV-1 inhibits the nuclear translocation of NF-κB subunits. These two proteins lead to abrogated NF-κB activity.^117,118^

In addition to the process targeted by HSV-1, other key signalling transducers of the cGAS-STING pathway have been reported to be antagonised by certain pathogens. For instance, the vaccinia virus utilises a nuclease, named poxvirus immune nuclease or poxin, to specifically hydrolyse 2′,3′-cGAMP, therefore disconnecting cGAS and STING.^119^ Similarly, CdnP encoded by *Mycobacterium tuberculosis* degrades both the bacterial c-di-AMP and host 2′,3′-cGAMP, leading to reduced levels of STING agonists.^120^ The murine cytomegalovirus protein m152 specifically targets the type I IFN response by binding to STING, thereby delaying its trafficking to the Golgi compartment.^28^

Overall, multiple proteins encoded by pathogens that attenuate the cGAS-STING pathway fully illustrate the high selective pressure on pathogens imposed by host immunity. Intriguingly, while NF-κB signal transduction was shown to be inhibited by certain viruses, some viruses seem to actively utilise the NF-κB pathway to promote pathogenesis.^121–124^ Several strategies to subvert or exploit autophagy were identified to be utilised by various bacterial pathogens (reviewed in ref.^125^) These phenomena suggest that pathogens not only “fight with” the host but may also “cooperate with” it for their own benefits.

### Avoidance of DNA exposure

Viruses can also prevent the activation of the cGAS-STING pathway in the host by simply shielding their DNA from recognition by cGAS. Despite the multiple sensing strategies adopted by the cGAS-STING pathway to counter viruses (such as the establishment and amplification of immunity in bystander cells, indirect pathogen detection through sensing self-DNA, and so on, as mentioned above), the sensitivity of the pathway must be carefully restricted to avoid untoward consequence as a result of autoimmunity. Common restrictions on cGAS-STING pathway activity include limited concentration of cGAS in the cytosol under resting states, inhibitory PTMs, and so on.^73,85,98,99,102,103^ The restriction of pathway sensitivity is another strategy that allows pathogens to survive if they successfully shield their DNA, as is the case of HIV-1.

HIV-1 initiates the reverse transcription of its genomic RNA into dsDNA shortly after entering its target cells (e.g. CD4^+^ T cells, dendritic cells and macrophages), an activity that might be expected to trigger innate PRR.^126–130^ Before integration, HIV-1 reduces DNA exposure to achieve evasion from surveillance by cGAS or other cytosolic DNA sensors.^129–132^ Following envelope-mediated fusion, HIV-1 associates with the cellular microtubule system through the dynein adaptor protein bicaudal D2 (BICD2) via viral capsids to facilitate its trafficking to the nucleus. The depletion of BICD2 leads to increased innate sensing of HIV-1 infection, which might result from the cytoplasmic accumulation of HIV-1 cDNA and increased recognition of cDNA by cGAS.^133^ Further, HIV-1 capsids also recruit the host protein cleavage and polyadenylation specific factor 6 (CPSF6) and cyclophilins to attenuate viral DNA synthesis in the cytosol and facilitate viral DNA nuclear entry, therefore preventing viral DNA detection by cytosolic cGAS^134^ (Fig. 5). In addition, cellular antiviral factors, such as apolipoprotein B mRNA editing enzyme catalytic subunit 3 G (APOBEC3G), sterile alpha motif and HD domain-containing protein 1 (SAMHD1), and three prime repair exonuclease 1 (TREX1), further restrict the amount of viral cDNA in the cytosol, which practically limits ligands for cGAS.^134–137^ Therefore, HIV-1 exploits host factors to reduce the exposure of its viral cDNA to cytosolic cGAS, thus rendering the invasion process almost ‘silent’ and successfully colonising the infected cells.

## Targeting the cGAS-STING pathway for antimicrobial therapies

Given its potent antimicrobial capacity, it is attractive to consider intervening in the cGAS-STING pathway for therapies against pathogens. Chitosan, as a candidate vaccine adjuvant, was proved to trigger type I IFN secretion, dendritic cell maturation and T helper type 1 (Th1) cell responses. The underlying mechanism might be related to the chitosan-induced mitochondrial stress and subsequent release of mtDNA, which then trigger the activation of the cGAS-STING signalling.^138^ In addition, cGAMP was suggested to be a potential adjuvant due to its capability to enhance antigen-specific antibody production and T-cell responses in mice.^4,139^ Encapsulating cGAMP into cationic liposomes or endosomolytic polymersomes further elevates the efficiency of cGAMP by overcoming its poor membrane permeability.^140,141^ Recently, cGAMP encapsulated in pulmonary surfactant (PS-cGAMP) has been shown to be an effective adjuvant for influenza vaccines in mice and ferrets.^142^ This vaccine induces robust cross-protection against a wide range of influenza virus subtypes within 2 days, lasting for 6 months, without overt lung inflammation.^142^ In addition, blocking STING degradation after its activation using bafilomycin A1 (BafA1) reportedly promotes cGAMP-mediated immune responses both in vitro and in vivo, which represents a novel method to modulate the cGAS-STING pathway.^143^

However, considering that some pathogens encode proteins counteracting the downstream signalling of the cGAS-STING pathway, generally stimulating the cGAS-STING activity may not be effective in some contexts. Therefore, antagonising pathogen evasion mechanisms provides another strategy to magnify antimicrobial responses. For example, the administration of inhibitors of the bacterial phosphodiesterase CdnP, which is encoded by *M. tuberculosis* to evade the cGAS-STING pathway through the hydrolysis of cGAMP, reduces bacterial pathogenicity.^120^ Utilising drugs that specifically target viral molecules might also avoid the potentially detrimental consequence of the overactivation of the cGAS-STING pathway.

In addition, as the different outcomes of the activated cGAS-STING pathway might exert distinct influences on pathogens, the development of drugs that can direct the cGAS-STING signalling to antimicrobial outcomes but not to promicrobial outcomes should be considered. Overall, a more precise intervention in the cGAS-STING pathway would certainly promise more effective and secure therapy methods, but it also calls for a deeper understanding of the interactions between pathogens and the cGAS-STING pathway.

## cGAS-STING pathway in cancer

Beyond the well-known role of the cGAS-STING pathway in combating pathogens, recent studies have also revealed the role of this pathway in cancer. Many cancer cells present genome instability, which leads to the appearance of cytosolic DNA and activation of cGAS. Therefore, the cGAS-STING pathway may serve as a pivot linking cancer and immunity, which triggered researchers to explore the role of this pathway in cancer development and its potential in cancer therapies.

In cancer cells, genome instability results in the formation of micronuclei in a cell-cycle-dependent manner. The rupture of the micronuclear envelope exposes the genome DNA to cytosolic cGAS, which leads to the activation of cGAS.^70,144^ Furthermore, in cancer cells with mitochondrial dysfunction, the release of mitochondrial dsDNA serves as an agonist for cGAS.^145^ The cGAS-STING pathway is also activated in immune cells. Tumour-derived exosomes have been suggested to deliver tumour DNA to nearby dendritic cells.^146^ The breakdown of the micronuclear membrane might represent a mechanism to transport tumour DNA into the exosomes.^147^ In addition, immune cells are also suggested to receive tumour-derived cGAMP through gap junctions or cGAMP transporters, therefore leading to the activation of STING signalling.^81,82,148–150^

The activation of the cGAS-STING pathway results in either antitumour or protumorigenesis processes, depending on the context. On the one hand, cytokines, such as type I IFN, induced by the activated cGAS-STING pathway boost natural killer (NK) cell responses and prime CD8^+^ T cells for a more potent tumour surveillance.^148,151^ In addition, the activation of the cGAS-STING pathway leads to the induction of a set of senescence-associated secretory phenotype (SASP), which amplifies cell senescence, thereby restricting tumourigenesis.^152,153^ On the other hand, the activation of cGAS and STING has been linked to metastasis and immune evasion in cancers. It has been reported that tumour cells presenting high genome instability, which is the hallmark of metastatic tumours but not of primary tumours, utilise the chronic activation of the cGAS-STING pathway to facilitate cellular invasion.^154^ Notably, this invasion is mediated by STING-dependent noncanonical NF-κB signalling, whereas canonical NF-κB signalling and type I IFN responses are associated with better prognosis. In another study, researchers have reported that the transport of brain tumour-derived cGAMP to astrocytes via gap junctions induces the secretion of IFNα and TNFα in a STING-dependent manner. The inflammatory cytokines activate the STAT1 and NF-κB pathways in brain tumour cells, promoting metastasis and chemoresistance.^150^ Furthermore, cGAS and STING have been reported to participate in shaping the immune-suppressive tumour microenvironment by recruiting regulatory T cells and myeloid suppressor cells, as well as upregulating immunosuppressive proteins, such as programmed death ligand 1 (PD-L1) and C–C motif chemokine receptor 2 (CCR2), thereby promoting tumour immune evasion.^155–157^ The detailed mechanism leading to these distinct outcomes of the activation of the cGAS-STING pathway in cancer remains poorly understood. Further investigation of the molecular details of the cGAS-STING pathway in different types, stages, and microenvironments of cancers might help to address this knowledge gap.

Promising results have been achieved by utilising STING agonists in cancer therapies. These therapeutic effects are largely explained by the priming of CD8^+^ T cells and activation of NK cells in antitumour responses. Therefore, this might represent a potent strategy against immune checkpoint inhibitor-resistant cancers due to the lack of antitumour T-cell responses and against major histocompatibility complex I (MHC-I)-deficient tumours, which evade T-cell surveillance.^158,159^ This strategy has achieved better outcomes through several improvements, such as the addition of modifications to CDN analogues, envelopment of CDNs in liposomes or nanoparticles, and combination with programmed cell death protein 1 (PD-1) blockage, that aimed to increase the stability and lipophilicity of the agonists and their affinity to hSTING and to facilitate the systemic delivery of the agonists.^156,158–160^ Two phase I clinical trials with STING agonists (ADUS100 and MK1454) have received good feedback, showing that dose escalation was tolerated and that CD8^+^ T-cell infiltration in tumours was evident.^161,162^ However, both STING agonists showed maximum efficacy only when delivered intratumourally, which might limit their application to accessible tumours. Researchers have also reported agonists of STING that are not derived from CDNs but are amidobenzimidazole derivatives.^163,164^ Amidobenzimidazole derivatives are amenable to intravenous administration and therefore might be able to initiate immune responses towards multiple heterogenous, distal tumours. Notably, the administration of STING agonists to patients with cancer might also lead to immune evasion of cancer cells and even aggravate metastasis as mentioned above. Therefore, understanding the factors that dictate the consequences of cGAS-STING pathway activation in different contexts is essential to ensure satisfactory clinical outcomes.

## Conclusions and future perspectives

In the past few years, exciting structural, genetic, and biomedical studies have dramatically deepened our understanding of the molecular mechanisms underlying the canonical cGAS-STING-IRF3 axis.^18,24,26,27^ However, much remains to be explored regarding the mechanisms of the remaining inputs and outputs of cGAS-STING, as well as their contribution to pathogen detection and elimination. Therefore, future studies are needed to fully illustrate how the cGAS-STING pathway has gained multiple functions in the fight against pathogens during its long course of evolution. Furthermore, the significance of species-specific characteristics of the cGAS-STING pathway in the defence against pathogens is also an interesting field to be explored. More profound appreciations of the evolution of the cGAS-STING pathway would provide us with insights into the strategies used against pathogens from the perspective of pathway plasticity.

Recent discoveries regarding the regulation of the cGAS-STING signalling pathway during infections have demonstrated its capability to integrate multiple intracellular and extracellular information to guarantee a sensitive detection of pathogen invasion. However, how this sensitivity is carefully managed to avoid detrimental consequences of hypersensitive immune responses is still not well understood. Our understanding of the dynamic regulation of the cGAS-STING pathway during infections is still at its infancy. Although multiple enzymes responsible for the PTMs of cGAS and STING have already been identified, how the different kinds of PTMs interact with each other, as well as how the activities of these enzymes are timely regulated, is largely unknown. More in-depth studies on the PTMs or other regulations of the cGAS-STING pathway may open new perspectives on how the host manipulates this pathway in response to dynamic infection processes.

The counteractions of pathogens against the cGAS-STING pathway have also been identified, with several counteraction mechanisms being already elucidated. Whether (and how) the evasion strategies that pathogens adopt vary with the cell context (e.g. cell types and cell phases) and how these evasion mechanisms develop remain to be explored. A better understanding of the counteraction strategies adopted by pathogens would deepen our knowledge on how the host deals with these evasion mechanisms, thereby enabling us to develop more effective and safer antimicrobial drugs.

Finally, recent studies have also shed light on the roles of the cGAS-STING pathway in cancers. The activation of cGAS and STING might exert either positive or negative influences on cancer development, depending on the context. Further studies on the molecular mechanisms of this pathway under different contexts might lead to a better prediction of the outcomes of its activation, which would enable us to take advantage of the cGAS-STING pathway in cancer therapies.

In summary, in recent years, we have witnessed a significant expansion of the knowledge on the interactions between the cGAS-STING pathway and pathogens, as well as on the role of this pathway in cancers. Further studies in this field are necessary to improve antimicrobial and antitumour therapies.
